# Postglacial Colonization of the Qinling Mountains: Phylogeography of the Swelled Vent Frog (*Feirana quadranus*)

**DOI:** 10.1371/journal.pone.0041579

**Published:** 2012-07-25

**Authors:** Bin Wang, Jianping Jiang, Feng Xie, Cheng Li

**Affiliations:** Chengdu Institute of Biology, the Chinese Academy of Sciences, Chengdu, China; American Museum of Natural History, United States of America

## Abstract

**Background:**

The influence of Pleistocene climatic fluctuations on intraspecific diversification in the Qinling–Daba Mountains of East Asia remains poorly investigated. We tested hypotheses concerning refugia during the last glacial maximum (LGM) in this region by examining the phylogeography of the swelled vent frog (*Feirana quadranus*; Dicroglossidae, Anura, Amphibia).

**Methodology/Principal Findings:**

We obtained complete mitochondrial *ND2* gene sequences of 224 individuals from 34 populations of *Feirana quadranus* for phylogeographic analyses. Additionally, we obtained nuclear tyrosinase gene sequences of 68 *F. quadranus*, one *F. kangxianensis* and three *F. taihangnica* samples to test for mitochondrial introgression among them. Phylogenetic analyses based on all genes revealed no introgression among them. Phylogenetic analyses based on *ND2* datasets revealed that *F. quadranus* was comprised of six lineages which were separated by deep valleys; the sole exception is that the Main Qinling and Micang–Western Qinling lineages overlap in distribution. Analyses of population structure indicated restricted gene flow among lineages. Coalescent simulations and divergence dating indicated that the basal diversification within *F. quadranus* may be associated with the dramatic uplifts of the Tibetan Plateau during the Pliocene. Coalescent simulations indicated that Wuling, Daba, and Western Qinling–Micang–Longmen Mountains were refugia for *F. quadranus* during the LGM. Demographic analyses indicated that the Daba lineage experienced population size increase prior to the LGM but the Main Qinling and the Micang–Western Qinling lineages expanded in population size and range after the LGM, and the other lineages almost have stable population size or slight slow population size decline.

**Conclusions/Significance:**

The Qinling–Daba Mountains hosted three refugia for *F. quadranus* during the LGM. Populations that originated in the Daba Mountains colonized the Main Qinling Mountains after the LGM. Recent sharp expansion of the Micang–Western Qinling and Main Qinling lineages probably contribute to their present-day secondary contact.

## Introduction

Climatic changes associated with Pleistocene glacial cycles are believed to have caused montane species to shift, expand, or contract along latitudinal or elevational gradients [1]–[3]. Intraspecific populations adapted to different mountains may have experienced isolations and connectivity as well as range contractions and expansions following climate fluctuations; thus, prolonged isolation may led to substantial genetic heterogeneity among populations and long-term connectivity may contributed to dispersal or ongoing gene flow among mountains; and accompanying with these processes, the population size would be predicted to fluctuate [4]–[6]. The level of divergence together with ecological and biological factors usually affects the formation of independent lineages and whether populations occurring from different regions mix into a same gene pool when they have secondary contact [6]–[9].

Nevertheless, the diversification patterns influenced by Pleistocene climate cycles may have varied from place to place [7]–[9]. Many Europe and North American organisms retreated to the southern regions during the ice ages and recolonized the original north range during the interglacial periods [7]–[12]. In contrast, East Asia was probably characterized by a mosaic of mountains and likely experienced a relatively mild Pleistocene climate [13]–[15], a combination that probably provided stable habitats [16] and glacial refugia for species [17] throughout their entire ranges rather than species being confined only to southern latitudes within their present-day ranges [2], [3], [6]. In consistent to this speculation, some bird species probably persisted in its present range including the Qinling–Daba Mountains and southern China mountains south of Yangtze River during the glacial periods and then expanded during the warming time [18], [19], whereas some herpetology species probably hid into multiple refugia including the south Korea and the regions south of Yangtze River in the southern China during the ice ages [20], [21]; but most of them have probably expanded on population size and range during glacial periods before the last glacial maximum (LGM) [18]–[19]. So far, our knowledge on the influence of the Pleistocene climate changes affecting diversification of the East Asian species is limited to few literatures [18]–[21]; thus, further investigations on phylogeography and demography of the montane species in this region are necessary for testing above distinct hypotheses.

**Table 1:** Sampling information and haplotypes based on *ND2* gene for 34 sampled populations of *Feirana quadranus*.

Population	Location	n	Coordinates	Haplotypes
SZ	Sangzhi Co., Hunan Prov.	5	N29.6346°, E109.9232°	H1(2), H2(2), H3(1)
CY	Changyang Co., Hubei Prov.	10	N30.5853°, E110.9023°	H4(1), H5(2), H6(1), H7(1), H8(4), H9(1)
LC	Lichuan Co., Hubei Prov.	4	N30.5244°, E109.0946°	H17(4)
FJ	Fengjie Co., Chongqing City	5	N30.6168°, E109.4298°	H20(4), H21(1)
FA	Fangxian Co., Hubei Prov.	1	N31.9250°, E110.3231°	H24(1)
SN	Shennongjia, Hubei Prov.	1	N31.8211°, E110.5101°	H24(1)
WS	Wushan Co., Chongqing City	3	N31.3721°, E109.9074°	H26(2), H21(1)
WX	Wuxi Co., Chongqing City	7	N31.4804°, E109.9023°	H21(3), H24(1), H25(2), H27(1)
CK	Chengkou Co., Chongqing City	24	N32.0084°, E108.5349°	H13(1), H14(1), H15(1), H16(1), H18(1), H19(3), H28(1), H29(2), H30(1), H34(4), H35(7), H36(1)
KA	Kaixian Co., Chongqing City	13	N31.4051°, E107.8802°	H10(1), H11(7), H12(2), H22(1), H23(2)
LG	Langao Co., Shananxi Prov.	3	N32.1805°, E108.9288°	H30(1), H35(2)
ZB	Zhenba Co., Shananxi Prov.	3	N32.5774°, E107.9339°	H31(1), H32(2)
WY	Wanyuan Co., Sichuan Prov.	3	N32.0877°, E108.2387°	H17(1), H33(1), H35(1)
SY	Shanyang Co., Shananxi Prov.	7	N33.6501°, E109.9674°	H51(2), H58(5)
ZS	Zhashui Co., Shananxi Prov.	14	N33.7837°, E108.8367°	H55(1), H56(11), H57(1), H58(1)
CA	Chang’an Co., Shananxi Prov.	1	N33.7628°, E108.7731°	H46(1)
NS	Ningshan Co., Shananxi Prov.	12	N33.5482°, E108.5425°	H38(8), H44(1), H45(1), H47(1), H56(1)
FP	Foping Co., Shananxi Prov.	11	N33.6986°, E107.9491°	H38(8), H39(1), H40(1), H41(1)
TB	Taibai Co., Shananxi Prov.	3	N34.0573°, E107.5421°	H51(3)
ZZ	Zhouzhi Co., Shananxi Prov.	4	N33.7747°, E107.9742°	H38(2), H42(1), H43(1)
LX	Longxian Co., Shananxi Prov.	1	N34.9332°, E106.5798°	H53(1)
FX	Fengxian Co., Shananxi Prov.	18	N34.0983°, E106.5649°	H51(10), H70(7)
HX	Huixian Co., Gansu Prov.	25	N33.8963°, E105.8702°	H48(1), H49(1), H51(3), H63(1), H64(1), H65(6), H70(10), H71(1), H72(1)
LD	Liangdang Co., Gansu Prov.	6	N34.0021°, E106.3042°	H51(4), H70(2)
LB	Liuba Co., Shananxi Prov.	5	N33.7031°, E107.0848°	H51(4), H54(1)
YX	Yangxian Co., Shananxi Prov.	6	N33.5943°, E107.5387°	H37(1), H38(3), H50(1), H52(1)
LY	Lueyang Co., Shananxi Prov.	4	N33.2266°, E106.4017°	H70(4)
KX	Kangxian Co., Gansu Prov.	2	N33.2704°, E105.4367°	H70(2)
NZ	Nanzheng Co., Shananxi Prov.	4	N32.8446°, E106.8261°	H67(4)
NJ	Nanjiang Co., Sichuan Prov.	6	N32.5883°, E106.6750°	H66(1), H68(1), H69(4)
WE	Wenxian, Gansu Prov.	6	N32.7354°, E105.1841°	H61(3), H62(3)
QC	Qingchuan Co., Sichuan Prov.	2	N32.5778°, E104.7540°	H61(2)
BC	Beichuan Co., Sichuan Prov.	1	N31.7950°, E104.1262°	H60(1)
AX	Anxian Co., Sichuan Prov.	4	N31.7116°, E104.2656°	H59(4)

The Qinling–Daba Mountains in the central China comprise of series of mountains, such as Qinling, Daba, Micang, Longmen, and Wuling Mountains, that are delimited by hydrological systems (e.g. Jialing River, Han River and Yangtze River). Moreover, this region supports environments that are nearly unique among those for Oriental and Palearctic organisms that occur in temperate and sub-tropical ecosystems and are inhabited by considerable endemic species [17], [22]. Although most mountains were not glaciated during the Pleistocene [23]–[25], this region experienced climatic fluctuations that likely impacted species distributions, diversification and demography [17]. The frog group *Feirana*, belonging to the family Dicroglossidae, as stream-dwelling, inhabits this region and is highly dependent on mesic environments that restrict the distributions of them [26]–[31]. Therefore, it was easily affected by the Pleistocene climate changes.

The swelled vent frog *Feirana quadranus* [26] is restricted to montane streams throughout the Qinling–Daba Mountains [27]–[31]. Because of its distribution, phylogeographic study of this species may contribute to understanding the effects of Pleistocene climate changes on diversification in the Qinling–Daba Mountains. For example, in this species, populations on different mountains could derive from one refugium during the LGM, or may be restricted in multiple refugia and experience long–term vicariances. In the latter speculation, it may be predicted that populations on different mountains could probably merge into a common gene pool during the period of secondary contact. However, based on *12S*, *16S* and *ND2* gene sequences from limited number of samples, Wang et al. [28] indicated that *F. quadranus* was comprised of several divergent lineages restricted to different mountains and guess that these lineages have possibly experienced prolonged vicariances.

Therefore, in this study, we more intensively explore the phylogeography of *F. quadranus* based on *ND2* gene sequences to test above hypotheses concerning the diversification of this species. First, we use phylogenetic methods to test whether populations on different regions are reciprocally monophyletic. Second, we conduct multiple analyses to test if the identified lineages are isolated by barriers with unsuitable environments that restrict gene flow among them. Third, we use coalescent simulations to test for above alternative refugia hypotheses. Finally, we test whether historical population size fluctuations of lineages were related to the Pleistocene climatic fluctuations.

## Materials and Methods

### Sampling and Laboratory Protocols

**Figure 1:**
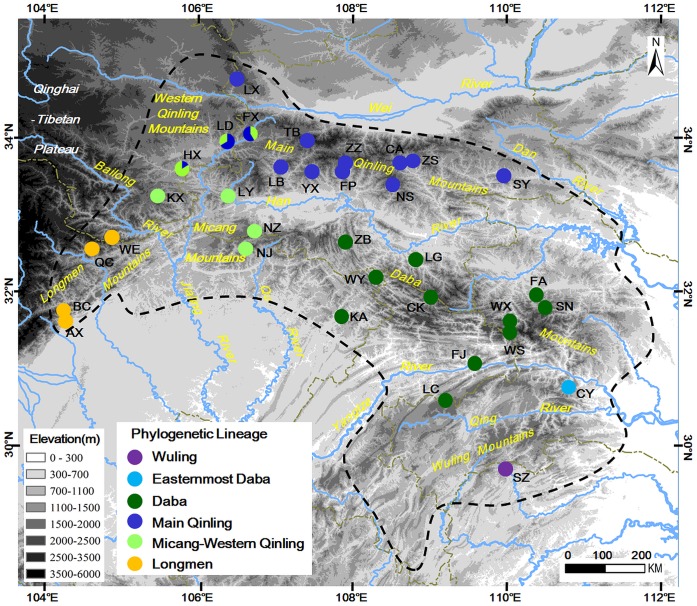
Locations of the 34 sampled populations of *Feirana quadranus*. Details of the populations are given in Table 1, and the phylogenetic lineages are given in Figure 3.

In total, we used 224 samples from 34 populations that cover the entire geographic range of *Feirana quadranus* (Table 1; Figure 1); data for eighteen samples derives from [28] (GenBank Accession No.: GQ225934, 37, 39, 41–50, 52, 53, 55, 56, 58). Voucher specimens were deposited in Chengdu Institute of Biology (CIB), the Chinese Academy of Sciences (CAS).

Total genomic DNA was extracted from muscle tissues (preserved in 95% Ethanol) using a standard phenol-chloroform extraction procedure [32]. To evaluate the presence and extent of mitochondrial introgression between *Feirana quadranus* and closely related species (*F. kangxianensis* newly published [33], and *F. taihangnica*), we amplified the nuclear tyrosinase gene sequences for 68 individuals from 28 localities of *F. quadranus*, one sample of *F. kangxianensis* and four samples of *F. taihangnica* from three localities (Table S1) using primers Tyr1G and Tyr1B [34]. PCR amplification and sequencing were performed according to procedures in other studies [34], [35]. We then obtained mitochondrial *ND2* gene sequences for all new samples used in this work according to the methods in our previous study [28]. Following other studies [28], [34], [36], [37], we used *Nanorana pleskei*, *Gyndrapaa yunnanensis*, *F. kangxianensis*, and *F. taihangnica* as outgroups. Data from these taxa were either sequenced in this present study or downloaded from GenBank (HQ324232, EU979962, EU979976, GQ225989, GQ225994–225996 and GQ226000). New sequences were deposited in GenBank (GenBank Accession No.: JX263025–JX263230, JX263231–JX263298 and JX263299–JX263302).

All sequences were aligned using MEGA 5.0 [38] and manually checked through examining both nucleotide and amino acid sequences to confirm nuclear homologs of target genes. Alignments were unambiguous and no indels were found in these two protein-coding regions. Because the present tyrosinase gene dataset is insufficient for adequately revealing intraspecific relationships (see the results), it was only used to identify whether *F. quadranus* is an independent evolutionary lineage distinct from *F. kangxianensis* and *F. taihangnica*. *ND2* gene sequences were used for phylogeographic study of *F. quadranus* as well as mitochondrial DNA (mtDNA) is commonly used to investigate historical evolutionary patterns [39]–[42].

### Phylogenetic Analyses

Haplotypes for our samples were identified by DNASP 5.1 [43], and subsequently used to reconstruct the gene genealogy using maximum parsimony (MP), maximum likelihood (ML) and Bayesian inference (BI), as implemented in PAUP 4.0b10 [44], PHYML 3.0 [45] and MRBAYES 3.0b4 [46], respectively. MP analyses were performed used same parameters as in [28]. For ML and BI analyses, the TrN + G model was selected for all datasets by likelihood ratio tests either under the Corrected Akaike Information Criterion (AICc) [47] or under the Bayesian Information Criterion (BIC) implemented in JMODELTEST 0.1.1 [45], [48]. In ML analyses, the values of the shape parameter of the Gamma distribution were set as 0.186 and 2.032 (estimated by JMODELTEST) for the *ND2* and tyrosinase datasets, respectively. The base frequency and ratio of transitions/transversions were optimized by the maximum likelihood criterion in PHYML. The default tree search approach using simultaneous Nearest Neighbor Interchange (NNI) method and BioNJ tree as starting tree was used to estimate tree topologies. Non-parametric bootstrapping with heuristic searches of 10000 replicates was used to assess confidences of branches in MP and ML trees [49]–[52]. In BI, according to the estimations of JMODELTEST, the value of gamma shape was set as 0.186 and the base frequencies was specified as (0.2985 0.3068 0.0954 0.2993); then we initiated two dependent runs each with four simultaneous Monte Carlo Markov chains (MCMC) for 50 million generations with sampling every 1000 generations and discarded the first 10% of generations as “burn-in”. The convergence of chains was confirmed until average standard deviation of split frequency is below 0.01, and the posterior probabilities (pp) were achieved from the remaining trees [53], [54].

### Population Structure Analyses

Haplotype network were constructed using parsimony method in the software TCS 1.21 [55] based on *ND2* sequences. For mtDNA analyses, DNASP 5.1 was used to compute the number of haplotypes (*H*), haplotypic diversity (*Hd*), and nucleotide diversity (*π*). To test whether there is significant geographical divisions and population genetic structure within this species, an analysis of molecular variance (AMOVA) was implemented in ARLEQUIN 3.1 [56]. The fixation indices *F*ct, *F*sc and *F*st that described variation among groups, variation within groups and the variation within populations, respectively, are estimated, and the most probable geographical subdivision were inferred with the highest value of *F*ct [56]. The significance of these statistics was determined by 10,000 permutation replicates.

To test the isolation by distance model (IBD) with a significant correlation between geographic and genetic distance (*F*st/1-*F*st), a Mantel test (10,000 randomizations) was performed in the web-based computer program IBDWS 3.14 [57]. Additionally, to highlight geographical discontinuity corresponding to breaks of gene flow among groups and to test if these boundaries are corresponding to major valleys in the Qinling–Daba Mountains (Figure 1), Monmonier’s maximum difference algorithm [58] was implemented in the software BARRIER 2.2 [58]. In this kind of analyses, geographical coordinates were used for each locality and connected by Delauney triangulation using a pairwise Tamura-Nei 93 genetic distance matrix for all populations; then the boundaries (the areas where differences between pairs of populations are largest) were identified using Monmonier’s maximum difference algorithm [58].

### Divergence Time Estimates

Under a ‘relaxed molecular clock’ assumption [59], BEAST 1.6.1 [60] was used to estimate the divergence dates among lineages. Under the TrN + G model (estimated by JMODELTEST), genealogy was reconstructed with an uncorrelated lognormal tree prior with an exponential growth prior and a lognormal mean rate (0.65% change per lineage per million years) of *ND2* gene which was inferred among previous studies of amphibians and reptiles [61]–[67]. For these analyses, two runs were conducted for 50 million generations with sampling every 1000 generations and in each run, 10% of the initial samples were discarded as burn-in. We checked the convergence of the chains through visual inspection of plotted posterior estimates and by determining the effective sample size (ESS) for each parameter sampled from the MCMC analysis using the program TRACER 1.5 [68]. The final tree including divergence estimates and their 95% highest posterior densities (HPD) were computed in TREEANNOTATOR 1.4.5.

### Historical Biogeography

To infer the distributional area of the most recent common ancestor (MRCA) of lineages, we used a ML method conducted in MESQUITE 2.75 [69]. In this analysis, six mountainous regions were regarded as separate character states and each *Feirana quadranus* individual was coded to one of these regions. The ancestral area was estimated under a ML model on the ML tree. Moreover, the Markov k-state 1 (Mk1) model with equal likelihood migration rates among mountains was selected because there is no prior information for dispersal rates. For each lineage, the area with the highest proportional likelihoods was suggested as the ancestral area.

**Figure 2:**
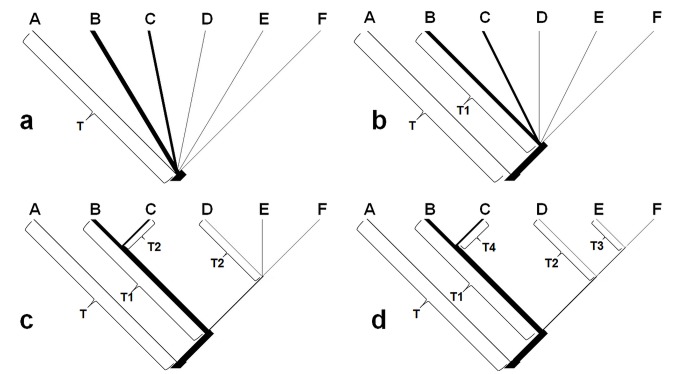
Models used to test refugial hypotheses for *Feirana quadranus* using coalescent simulations. Four hypotheses concerning the refugia during the last glacial maximum (LGM) were tested. a) single-refugium hypothesis, T = 50 ka (LGM is called the Dali glaciation in China); b) two-refugia hypothesis, T = 4.6 Ma, T1 = 50 ka; c) three-refugia hypothesis, T = 4.6 Ma, T1 = 3.3 Ma, T2 = 50 ka; d) multiple-refugia hypothesis, T = 4.6 Ma, T1 = 3.3 Ma, T2 = 2.1 Ma, T3 = 1.8 Ma and T4 = 0.5 Ma. The detail interpretation for these models is given in the text. Branch lengths are time in generations based on a 2.5-year generation time in *F. quadranus*. Branch widths (effective female population size, *N_ef_*) are scaled for each group based on the proportion of the total *N_ef_* that each group comprised. Groups A–E occurred from six mountainous regions were given in Figure 1.

Statistical phylogeography using coalescent simulations was performed to test the fit of the observed gene trees to phylogeographic hypotheses (see introduction) [8], [70]–[71]. In simulations, the grouping arrangement of populations was based on their geographical origins (Figure 1). Four hypotheses concerning refugia during the last glacial maximum (LGM) were tested for *Feirana quadranus* [72]. The first is the single-refugium model hypothesizing that all current populations were derived from a single refugium at the end of the LGM (Figure 2A). The second and the third hypotheses predicted that during the LGM there were either two or three refugia for *F. quadranus*, respectively (Figure 2B, 2C). The final is the multiple-refugia model that posits geographical groups experienced long-term vicariance and were isolated in multiple refugia (Figure 2D). Note that the long-term divergence among groups occurring before the LGM was estimated in this study using BEAST (see the results).

Two sets of coalescent simulations were conducted to test hypotheses using the software MESQUITE. In the first set, we simulated 1000 coalescent genealogies constrained within different hypothesized population trees and recorded the distribution of *S* [73], then through comparing the *S* value of the empirical ML genealogy with the *S* values of the simulated genealogies, we tested whether observed genealogies was consistent with the given hypotheses. In the second method, one hundred DNA sequence matrices were simulated under the optimal evolutionary model selected by JMODELTEST for observed dataset, then we reconstructed trees based on these matrices in PAUP and recorded the distribution of *S* values which were compared with *S* value of ML genealogy to test model fit. In all simulations, coalescent time (generations) was converted from absolute time (years) divided by 2.5 years as a generation time for *Feirana taihangnica* [29], [30].

Female effective population size (*N_ef_*) of each geographical group for coalescent simulations was converted from Theta using the equation *θ*  =  *N_ef_μ* with *μ* = 1.625E-8 (0.65E-8 * 2.5). The *θ*-values were estimated three times using ML method to ensure convergence in the program MIGRATE-N 3.3 [74] under the following parameters: 10 small chains for 200,000 steps and three long chains for 20 million generations with no heating and chains were sampled every 100 generations following a burn-in of 2 million generations. Total *N_ef_* were sum of the *N_ef_* for all groups and the proportion of total *N_ef_* that each group comprised were scaled the branch width of hypothesized trees (Figure 2) [75]–[77]. The 95% CI’s of *N_ef_* calculated from 95% CI’s of *θ*-values [78] were used as model parameters in simulations.

### Historical Demography

Deviations in Tajima’s *D* [79] and Fu’ *Fs* [80] implemented in ARLEQUIN were used to test a recent population expansion or bottleneck [79], [80]. The coalescent-based method Bayesian Skyline Plot (BSP) [81], as implemented in the software BEAST, was used to investigate population fluctuations of each lineage over time. The HKY model was selected by JMODELTEST for each lineage. The starting tree was randomly generated. Based on the number of samples, 3–10 grouped coalescent intervals (m) were prior chosen for different lineages; and the piecewise-constant model was selected as a prior skyline model. We conducted two MCMC runs for 20 million iterations, sampling genealogy and population size parameters every 1000 iterations and discarding the first 10% as burn-in. Additionally, we used the mean rate 0.65%/Ma to scale the time axis on BSP and uncorrelated lognormal model to account for rate variation among lineages. Convergence of the chains and ESS was also checked using TRACER.

## Results

### Phylogenetic Relationships

In the tyrosinase gene dataset (∼521 bps) including outgroup and ingroup sequences, 37 variable sites and 18 polymorphic sites were observed, but in ingroup sequences, we observed only 15 variable sites and 13 polymorphic sites. Twenty-five haplotypes were recognized for tyrosinase gene sequences of *Feirana quadranus*. Based on tyrosinase gene sequences, *F. quadranus* was revealed as monophyletic from closely related species (*F. kangxianensis* and *F. taihangnica*), but within *F. quadranus*, the relationships among haplotypes were reticular and not resolved.

**Figure 3:**
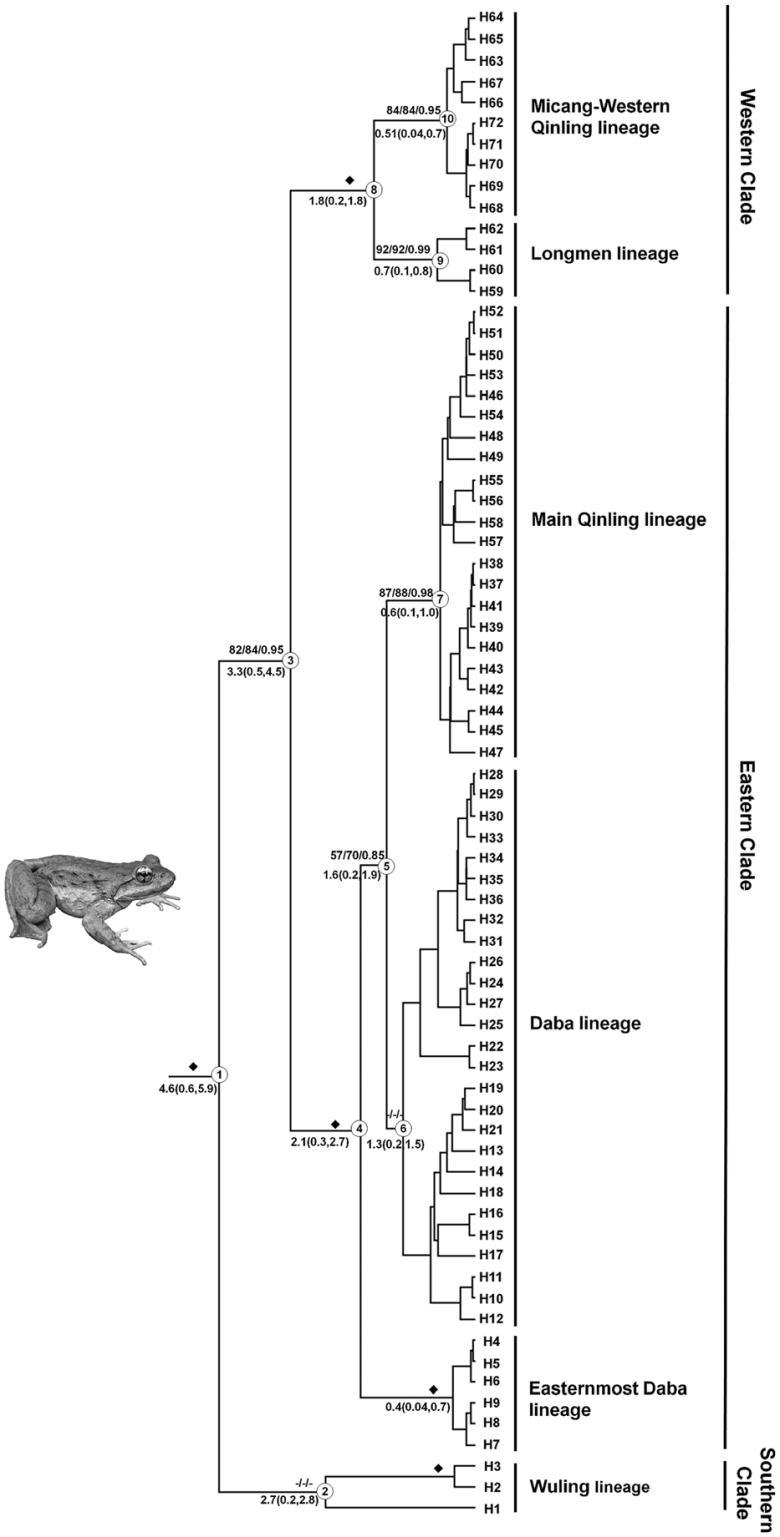
Bayesian tree for the 72 sampled haplotypes of *Feirana quadranus* based on *ND2* gene sequences. The bootstrap values calculated by Maximum Parsimony and Maximum Likelihood analyses and the Bayesian posterior probabilities from Bayesian analyses are presented above the main branches; the diamonds on branches mean that all support values are greater than 95%. The estimated divergence time (Ma, mean and 95% CI’s) for major nodes is below the branches. Ten major nodes (1–10) are indicated on the tree. Haplotypes (H1–H72) were given in Table 1.

An alignment of complete *ND2* gene sequences (∼1035 bps) was obtained for all samples and 72 haplotypes were recognized for all *Feirana* individuals (Table 1). MP analysis produced 1423 maximum parsimonious trees (403 steps; Consistency Index, CI = 0.8488; Retention Index, RI = 0.8036). The log-likelihood score of ML tree was -3097.60666. MP, ML and BI analyses revealed almost consistent topologies except for few tip branches with poor supports. All haplotypes of *F. quadranus* were clustered into a clade apart from other species (all supports  = 100%; Figure 3). *Feirana quadranus* is composed of three clades including six lineages (Figure 3). The first diverging clade, the Southern Clade, was only comprised of population WL from Wuling Mountains; however, its monophyly receives low supports (<50%; Figures 1, 3). The sister relationship of the Eastern and Western Clades was strongly supported (MP/ML/BI: 82/84/0.95). In the Eastern Clade, the basal lineage (Easternmost Daba lineage) includes only the population CY from the easternmost Daba Mountains, and the Daba lineage included populations from Daba Mountains, whereas the Main Qinling lineage was comprised of individuals from both the Main Qinling and Western Qinling Mountains (Figures 1, 3). Two lineages were found in the Western Clade. One lineage (Longmen lineage) included populations from the Longmen Mountains, and another lineage (Micang–Western Qinling lineage) included most populations from the Micang and Western Qinling Mountains and one population from Main Qinling Mountains (Figures 1, 3). Noticeably, each of populations FX, LD and HX meantime possessed haplotypes of Main Qinling lineage and Micang–Western Qinling lineage (Figure 1).

### Population Genetic Structure

**Figure 4:**
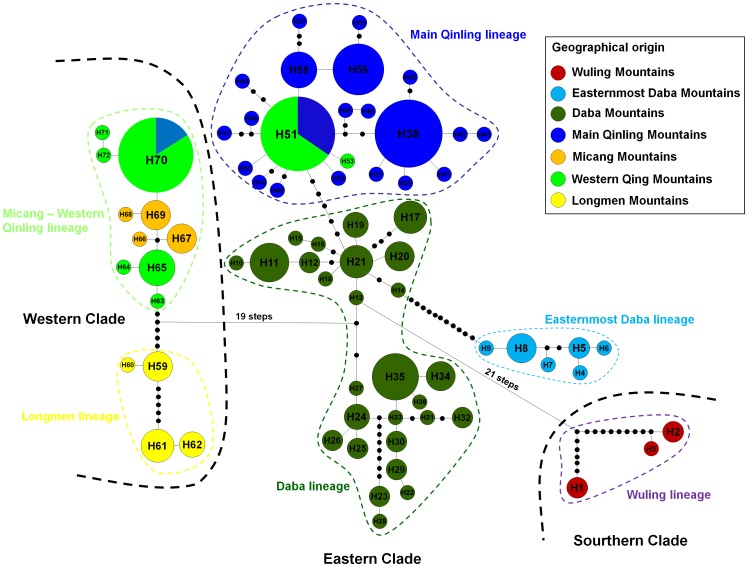
TCS network of the 72 sampled haplotypes of *Feirana quadranus* based on *ND2* gene sequences. Haplotypes (H1–H72) correspond to Table 1. The relative sizes of the circles in the network are proportional to the haplotype frequencies (n), and the black dots represent missing haplotypes. The phylogenetic clades and lineages were given in Figure 3.

TCS analysis based on *ND2* gene sequences yielded four unconnected haplotype groups with 95% confidence intervals (connect limit  = 14 steps; Figure 4). The Southern Clade connects with the Eastern Clade in 21 steps, and the Eastern Clade connects with the Western Clade in 19 steps. There is no common haplotype among three clades. In the Southern Clade, H1 is connected with H2 and H3 in 15 steps. In the Eastern Clade, the Easternmost Daba lineage connects only with Daba lineage in 11 steps, and the Daba lineage connects with Main Qinling lineage in five steps, but there is no common haplotype or population among these lineages. In the Western Clade, it is need five steps to connect the Micang–Western Qinling lineage with the Longmen lineage. Noticeably, haplotypes H51 and H70 meantime possess individuals from the Western Qinling Mountains and the Main Qinling Mountains and H53 occurring from the Western Qinling Mountains was nested into the Main Qinling lineage.

Substantial *Hd* was found for all lineages (0.66–0.95), but low *π* was found for all lineages (0.0016–0.0059) except the Wuling lineage (0.0097; Table 2). We tested the partitioning of genetic variation across four hypothesized sampling groupings according to geographical features and lineage relationships (Table 3; Figures 1, 3). Using AMOVA, significant genetic structure (P<0.01) was found across all hierarchical levels when all group allocations were tested (Table 3). These results rejected the null hypothesis that proposes no genetic structure existing within this species. Highest *F*ct (0.8117; P<0.001) was achieved when using grouping arrangement based on six phylogenetic lineages, and lowest *F*ct (0.5243; P<0.001) was showed when grouped all lineages into two large clades. Relatively, when all six lineages were grouped into three clades and five phylogenetic groups, high *F*ct (0.7182 and 0.7644; P<0.001) were also estimated.

**Table 2:** Genetic diversity and neutrality tests for phylogenetic lineages of *Feirana quadranus*.

Lineages (n)	*Hd*	*π*	Tajima’s *D*	Fu’s *Fs*
Wuling (5)	0.8000±0.1640	0.0097±0.006	1.6572	3.8033
Easternmost Daba (10)	0.8444±0.1029	0.0024±0.0016	−0.0893	−1.3403
Daba (67)	0.9498±0.0120	0.0059±0.0032	−0.8622	−7.8768*
Main Qinling (83)	0.8190±0.0266	0.0027±0.0016	−1.7820*	−10.0581**
Micang–Western Qinling (46)	0.6599±0.0721	0.0016±0.0011	−0.7854	−2.9476
Longmen (13)	0.7564±0.0698	0.0035±0.0021	1.5586	2.7607

Phylogenetic lineages were given in Figure 3. Mean ± standard deviation of *Hd* and *π* were given.

* P<0.05

** P<0.01.

**Table 3:** AMOVA analyses for different lineage arrangements of *Feirana quadranus*.

Lineage arrangement	Among groups *F*ct	Within groups *F*sc	Within populations *F*st
[1] [2] [3] [4] [5] [6]	0.8117	0.5158	0.9088
[1] [2] [3], [4] [5] [6]	0.7644	0.6989	0.9291
[1] [2], [3], [4] [5], [6]	0.7182	0.7598	0.9323
[1] [2], [3], [4], [5], [6]	0.5243	0.8834	0.9446

Lineages 1–6 were corresponding to Wuling, Easternmost Daba, Daba, Main Qinling, Micang–Western Qinling and Longmen lineages. P<0.01 for all AMOVA analyses.

The Mantel test resulted in an uncorrelated relationship (r^2^ =  −0.0784; P>0.05) between genetic distance (*F*st/1-*F*st) and geological distance. The barrier prediction analysis highlighted several putative barriers to gene flow that separate clades or lineages of *Feirana quadranus* (Figures 1, 3). The first predicted barrier corresponds to the Qing River that separates the Southern Clade from the Eastern Clade; the second is a barrier basically corresponding to the Qu River–upper Han River–part of the upper Jialing River that separates the Eastern Clade from the Western Clade; the third barrier basically corresponds to the Yangtze River separating the Easternmost Daba lineage from the Daba lineage; the fourth barrier is perfectly in line with the Bailong River separating the Longmen lineage from the Micang–Western Qinling lineage; and the fifth barrier is perfectly in line with the Han River which separates the Main Qinling lineage from the Daba lineage (Figure 1).

### Divergence Time, Ancestral Area and Hypotheses Testing

The divergences among lineages within *Feirana quadranus* were estimated as having occurred from Pliocene to the mid-Pleistocene (Figure 3). The results of the ancestral area reconstruction and divergence dating indicated that *F. quadranus* probably originated in the Wuling Mountains (Proportional likelihoods is 0.57) during the Miocene (Figure 3; Table 4). The divergence of the Southern Clade from other two major clades possibly occurred about 4.6 Ma (95% CI’s: 0.6–5.9 Ma), and that within this clade maybe occurred in the early Pleistocene (2.2 Ma, 95% CI’s: 0.2–2.4 Ma). The most recent common ancestor (MRCA) of Eastern and Western Clades was most probably in the Daba Mountains (Proportional likelihoods is 0.45), and these lineages approximately diverged about 3.3 Ma (95% CI’s: 0.5–4.5 Ma). Eastern Clade probably originated in the Daba Mountains (Proportional likelihoods is 0.96). In the early Pleistocene (about 2.1 Ma, 95% CI’s: 0.3–2.6 Ma), the Easternmost Daba lineage was possibly separated from the Daba and Main Qinling lineages whose MRCA maybe occurred in the Daba Mountains (Proportional likelihoods is 0.98). The MRCA of the Main Qinling lineage was in the Western Qinling Mountains (Proportional likelihood is 0.99) and maybe diverged from the Daba lineage during the Pleistocene (1.6 Ma, 95% CI’s: 0.2–1.9 Ma). The Western Clade probably originated in its present-day range (Proportional likelihood is 0.37 for each of Micang Mountains and Western Qinling Mountains), and the split between its two lineages (Micang–Western Qinling lineage and Longmen lineage) approximately occurred during the early Pleistocene around 1.8 Ma (95% CI’s: 0.2–1.8 Ma).

**Table 4:** Proportional likelihoods of the ancestral area for major lineages of *Feirana quadranus*.

Node	Proportional likelihoods					
	Wuling Mt.	Daba Mt.	Qinling Mt.	Western Qinling Mt.	Longmen Mt.	Micang Mt.
1	0.57	0.23	0.02	0.08	0.08	0.02
2	0.98	<0.01	<0.01	<0.01	<0.01	<0.01
3	0.21	0.45	0.02	0.15	0.15	0.01
4	0.01	0.96	<0.01	<0.01	<0.01	<0.01
5	<0.01	0.98	<0.01	<0.01	<0.01	<0.01
6	<0.01	0.99	<0.01	<0.01	<0.01	<0.01
7	<0.01	<0.01	0.99	<0.01	<0.01	<0.01
8	0.07	0.15	0.01	0.37	0.37	0.01
9	<0.01	<0.01	<0.01	<0.01	1	<0.01
10	<0.01	<0.01	<0.01	1	<0.01	<0.01

Nodes 1–10 were given in Figure 3. Mt. means Mountains.

Total *N_ef_*  = 2,043,076 (95% CI: 990,000–5,360,000) was achieved from an overall *θ* of 0.0332 (95% CI: 0.0162–0.0871) estimated for our *ND2* data. The coalescent times of the current trees were simulated under the ‘without migration’ model. The Slatkin and Maddison’s *S* for our ML genealogy was equal to 21. From both sets of coalescent simulations, the 95% CI’s of *S*-values of simulated trees (*S* >23) for the single-refugium model and two-refugia model did not include 21 (*S*-value of ML genealogy), indicating that these models were rejected (P<0.001), while the multiple-refugia model was also rejected (95% CI’s of *S*-values <6; P<0.001). However, the three-refugia model could not be rejected (95% CI’s of *S*-values were 19–28 including 21; P>0.05).

### Demographic History

Tajima’s *D* and Fu’s *Fs* statistics were positive for the Wuling and Longmen lineages (Table 3) suggesting either constant or contracting population size. The remaining lineages all possess negative Tajima’s *D* and Fu’s *Fs* values (Table 3) probably indicating that these lineages have undergone recent expansion. However, Fu’s *Fs* values were significant only for the Daba and Main Qinling lineages and Tajima’s *D* values were significant only for the Main Qinling lineage (Table 3). Noticeably, the power of Fu’s *Fs* was substantially affected by the number of segregating sites although its power was higher than mismatch distribution and Tajima’s *D* [82]. Therefore, Coalescent methods such as BSP taking genealogy into account may provide a better estimate of demographic history.

The effective sample size (ESS) for each parameter of the BSP was greater than 200, suggesting that the 20 million generations were sufficient to determine the demographic history for each examined lineage. The Wuling and Longmen lineages have maintained slightly slow population decline over time. The Easternmost Daba lineage and the Daba lineage have each experienced slow population growth after about 50,000 and 100,000 years ago, respectively; and at about 20,000 years ago, the Main Qingling and Micang–Western Qinling lineages began sharp expansion on population size. Noticeably, in the Holocene, the Wuling and Longmen lineages probably accelerated decreases in population size, while the Easternmost Daba lineage, Main Qingling lineage, and Micang–Western Qinling lineages maintained stable population sizes and the population size of the Daba lineage decreased. Through comparing recent population size fluctuations among lineages, we found that the Main Qinling lineage displayed the largest increase on population size (about 70-fold) whereas the Micang–Western Qinling lineage experienced about 20-fold population size increase over about past 17,000 years. The population sizes of other lineages changed within 10-fold.

## Discussion

Genetic breaks existing among intraspecific populations restricted to different mountains often correspond to low-elevation areas with warmer and drier conditions that form barriers restricting gene flow among populations [7], [12], [72], [76], [77], [83]. *Feirana quadranus* is restricted to montane streams in six major mountains separated by deep valleys or connected only by narrow passes in the Qinling–Daba Mountains (Figure 1). Accordingly, six divergent lineages structured across these mountains were revealed for *F. quadranus* (Figures 1, 3 and 4); meanwhile, hierarchical AMOVA (Table 3) and IBD test also indicated restricted gene flow among these lineages. Barrier analyses further highlighted the genetic interruptions between pairs of lineages that occur in the vicinity of the major valleys, such as those of the Qing River, Qu River, Bailong River, Jialing Rive and Han River (Figure 1). Although niche models were not tested for *F. quadranus*, the existence of putative low-elevation barriers with relative warmer and drier conditions suggest that niche conservatism probably plays an important role in driving diversification within this montane species [84], [85]. Exceptionally, some lineages presently span a short distance across some barriers (e.g., the Main Qinling and Micang–Western Qinling lineages span a short distance across the upper Jialing River and the Daba lineage spans a short distance across the Yangtze River; Figure 1). This supports the idea that these lineages has expanded and contracted their range in the past. The presence of haplotypes that span across barriers (e.g. H51 and H70; Figure 4) is probably attributable to recent migration rather than incomplete lineage sorting [76], [86]. This postulation is also supported by the reality that these haplotypes located in the interior position of phylogenetic tree (Figure 3). If so, it is understandable that the separated effects of some larger rivers (Han River, Jialing River and Yangtze River) are look like lower than that of those big tributaries (Bailong River, Qu River and Qing River). Considerable spatial genetic differentiations among lineages reflect the prolonged historical isolations among them even over Pliocene indicated by coalescent simulations. Thus, although there has been still no direct geological evidence for this phenomenon, we could guess that big tributaries probably have possessed longer geological history than the upriver of those stem river, indicating that these tributaries isolated populations longer than the upper stem rivers.

During Pliocene and Pleistocene, the rapid uplifts of the Tibetan Plateau are hypothesized to have induced environment shifts [87] that may have promoted diversification in the southeastern Asia [88]–[90]. The rapid uplifts of the Tibetan Plateau are believed to correspond to three phases (around 3.6 Ma, 2.6 Ma and 1.7 Ma, respectively) that are accompanied by the formation of the Asian monsoon, the beginning of Chinese loess, and appearance of the Yellow River [87], [91], although the time of origin of the current Asian monsoon is still debated [92], [93]. The geographical feature of Qinling–Daba Mountains at the eastern portion of the Tibetan Plateau was affected by these drastically movements [87], [94]. Divergence time estimations and coalescent simulations suggested that the divergence among three major clades within *Feirana quadranus* occurred approximately during this period (Figures 2 and 3). Consequently, we suggested that the basal diversification of this species were probably affected by the significant tectonic events associated with the rapid uplift of the Tibetan Plateau during the Pliocene–early Pleistocene.

**Figure 5:**
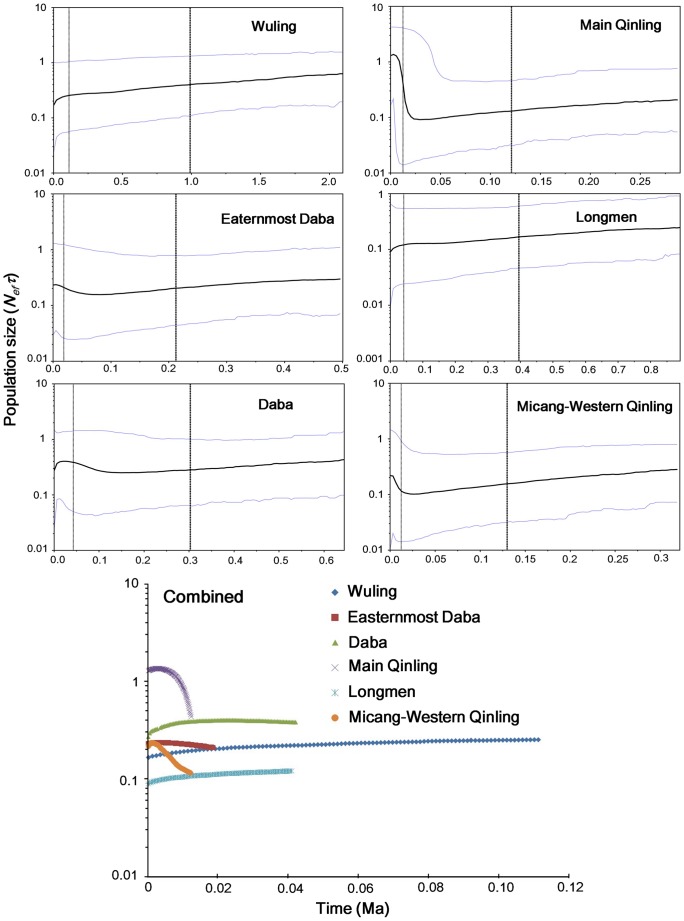
Demographic patterns of each lineage and a combined pattern as determined from BSP. The central solid line represents the median value for the log of the population size (*N_ef_* * τ) and the area between two thinner lines represent the 95% higher posterior density. The thicker dashed line represents the median of estimation time of the most recent common ancestor (MRCA), and the thinner is the lower limit of the 95% confidence interval. The combined figure shows the population size fluctuation after the lower limit of 95% CI’s of estimation time of the most recent common ancestor. Phylogenetic lineages correspond to Figure 3.

Historical allopatry and multiple refugia during the LGM have been suggested by previous studies of East Asian animals [18]–[21]. Through coalescent simulations for *Feirana quadranus*, we found three refugia located in the Wuling Mountains, Daba Mountains, and the Longmen–Micang–Western Qinling Mountains, respectively (Table 4; Figure 2C). The first two are consistently recognized as refugia for frogs [20], reptiles [21], and birds [19]. But the third region was firstly exactly recognized as a refugium for vertebrates using an effective method (coalescent simulations), although few studies indicated this point with no direct result as evidence [18], [19]; whereas, this is in accordance with expectations. As mentioned above, these regions possessed suitable environments for many temperate and subtropical species [15], [22]. During glacial periods, local climate changes in this region were probably ameliorated by orographic precipitation caused by the windward slopes of the Tibetan Plateau and Qinling Mountains though it is inland with weaker summer Asia monsoons. The moist condition in these regions during the last glacial period is also supported by palaeoclimatic evidence [14], [95]. It is conceivable that in these refugia *F. quadranus* survived in the lower elevations during the LGM, with subsequent dispersals (Figure 5). Of course, additional cases with congruent phylogeographic patterns will further reveal the legacies of glacial cycles for organisms in this region. Additionally, in contrast to phylogeographical studies on birds [18], [19] but consistent with those on the black-spotted frog [20] and short-tailed pit viper [21], we did not recover the Main Qinling Mountains as a refugium for the swelled vent frog. Instead, based on coalescent simulations and ancestral area reconstructions, we propose that populations presently holding this region originated from the Daba Mountains and colonized the Main Qinling Mountains and even some part of the Western Qinling Mountains after the LGM (Table 4). Colder conditions in the Main Qinling Mountains, including ice sheets in some mountains (e.g. Taibai Mountain) during the LGM [96], [97], possibly excluded this poikilotherm from this region, which originated in more southern areas. Moreover, the competition from its sibling species *F. taihangnica* which is native to the Qinling Mountains [28] had also probably contributed to the absence of this species in the Main Qinling Mountains during the ice ages.

The dispersal model also illustrates the demographic history and historical vicariance of this frog species. As one example, the secondary contact and overlapping ranges of haplotypes occurred between the Main Qinling lineage and the Micang–Western Qinling lineage is supported (Figures 3, 4). Two haplotypes (H51 and H53) representing individuals from four populations in the Western Qinling Mountains were resolved as parts of the Main Qinling lineage, as well as some individuals of haplotype H70 now occurs from the Main Qinling Moutains was nested into the Micang–Western Qinling lineage (Figure 4). The expansion and secondary contact presumably occurred after the LGM because of the relative interior position of these lineages and based on coalescent simulations. This hypothesis is supported by BSP analyses which reveal that the Main Qinling and Micang–Western Qinling lineages experienced sharp expansions during the present interglacial period in China (Figure 5) [98]. As another example, ancestral area reconstructions indicated that the ancestor of the Eastern Clade was in the Daba Mountains (Table 4), inferring that populations in the Main Qinling Mountains have dispersed from the Daba Mountains and invaded the Qinling Mountains after the LGM. This idea is also supported by the coalescent simulations and the polyphyletic relationships among lineages within the Eastern Clade (Figure 3). Based on the topography of this region, we suggest the hilly region north of the population ZB as the dispersal conduit for the ancestor of the Main Qinling lineage from the Daba Mountains to the Main Qinling Mountains, which is geographically distinct from the lower-elevation valley Han River (Figure 1). Currently, the absence of this frog in the vicinity of the Han River indicates that gene flow between the Daba and Main Qinling lineages has decreased. On the other hand, the biogeographic events of this species reflect that the Han River probably ran through the hilly region between Daba and Qinling Mountains after the LGM.

According to our mtDNA and nuclear genealogies, we found no hybridization between *F. quadranus* and its sister group, but we could not determine whether extensive gene flow via male dispersal occurred between mtDNA lineages within *F. quadranus* because nuclear sequences examined here provided only a small amount of information. If there is hybridization within *F. quadranus*, it is likely that lineages hybridize within the areas where they come into secondary contact, yet this small amount of hybridization is not enough to obscure historic effects of prolonged isolation among lineages [99], [100], and mitochondrial introgression likely does not extend much beyond contact zones. Still, it is desirable to examine extensive gene flow via male dispersal between lineages [101], [102] through deep studies based on multiple nuclear loci, which may either corroborate or overturn the observed mtDNA phylogenographic patterns of this species.

The repetitive geographic shifts and population size fluctuations in species associated with Pleistocene glacial oscillations are expected to be reflected by increases or decreases in levels of genetic variation and coalescence times [1]–[3]. In Europe and North America, species expanded from southern refugia after the LGM [103], and the genetic diversity of these species is usually geographically structured with declines in genetic diversity towards the north [1]. In this study, genetic diversity did not show a significant north–south trend among populations of *Feirana quadranus*, suggesting the existing of multiple refugia along the Qinling and Daba Mountains during the LGM. The significantly negative Fu’s *Fs* suggest recent expansion of the Main Qinling and Daba lineages and the BSPs consistently revealed population growth curves for these lineages, (Table 3; Figure 5). Differently, BSPs showed recent population increase but without significantly negative Fu’s *Fs* and Tajima’s *D* for Easternmost Daba and Micang–Western Qinling lineages. The interpretation for this discordance of neutral tests and BSPs could mean that populations underwent recent expansion but then were recently subdivided, subjected to substantial migration, and/or had experienced historical contractions [104]–[106]. Noticeably, the highest local genetic diversity was found in the Southern Clade (Wuling lineage) that includes only one population (Table 3). This could be partially attributed to historical contraction or subdivision evidenced by positive Tajima’s *D* and Fu’s *Fs* values [79], [80]. Similarly, the Longmen lineage exhibits relative low genetic diversity and positive Fu’s *Fs*, and Tajima’s *D* values (Table 3) that indicate that this lineage probably had experienced bottlenecks [79], [80] which was also supported by the BSP of this lineage (Figure 5). Of course, the limitation of sampling for these lineages probably affected our results; so we take a caution to make these speculations and would include more information such as sequences and genes to test these hypotheses in the future.

Some biological factors of amphibians, such as higher sensitivity to climatic changes [107], [108] and poor dispersal capability, probably restricted them into different refugia and experienced contractions during the LGM. The slight slow decline of the Wuling and Longmen lineages supports this speculation although BSPs could not deduce more long time coalescent events (Figure 5). The times of expansion among lineages look like different (Figure 5). The Daba and Easternmost Daba lineages probably expanded prior to the LGM (Figure 5) similar to the patterns observed for reptile and bird species in the southeastern China [18], [19], [21]. The interpretation of these previous studies was that the moderate climate after the Marine Isotope Stages (MIS) 16–18 [109] probably provided environments persistence through the glaciations. These studies suggested that the region in the southern part of Daba Mountains might be one refugium for these species [19]–[21], which also presumably supplied conditions for expansion of the Daba and Easternmost Daba lineages prior to the LGM. However, the sharp population size increases detected for the Main Qinling and Micang-Western Qinling lineages occurred after about 20 ka which is roughly consistent with the LGM during MIS2. Conceivably, the MRCA of Main Qinling lineage expanded significantly from the Daba Mountains at the end of the LGM. This speculation is also indicated by the coalescent simulations. As remarked above, during the glacial age, unfavorable environments in the Main Qinling Mountains north of the Daba Mountains and competition from closely related species had possibly restrained populations of *F. quadranus* from colonizing the Main Qinling Mountains.

## Supporting Information

Table S1 **Sampling information and haplotypes of** ***Feirana*** **based on nuclear tyrosinase gene.**
(XLS)
